# Two Molecular Subgroups Predict Most Recurrences in Advanced Laryngeal Squamous Cell Carcinoma

**DOI:** 10.1158/2767-9764.CRC-25-0249

**Published:** 2026-01-08

**Authors:** Todor M. Popov, Gergana S. Stancheva, Veronika Petkova, Silva Kyurkchiyan, Stoyan Dimitrov, Lyuben Dimitrov, Boryana Ilcheva, Albena D. Fakirova, Sylvia Skelina, Tzvetomir Marinov, Radka P. Kaneva

**Affiliations:** 1 Department of ENT, Medical University - Sofia, Sofia, Bulgaria.; 2 Molecular Medicine Center, Medical University – Sofia, Sofia, Bulgaria.; 3 Department of Pathology, Military Medical Academy, Sofia, Bulgaria.; 4 Department of Neurology, Medical University – Sofia, Sofia, Bulgaria.

## Abstract

The aim of this study was to test whether intratumoral heterogeneity explains inconsistent reports on VEGF-A as a prognostic marker in laryngeal squamous cell carcinoma (LSCC) and to identify expression phenotypes (mRNA/miRNA) associated with recurrence. In a prospective cohort of 60 patients with T3/T4 LSCC undergoing primary laryngectomy, we sampled four regions per case (tumor surface, tumor depth, peritumoral mucosa ≤1 cm, and paired distant normal mucosa). mRNA levels of hypoxia-inducible factor (HIF) 1α/2α/3α, VEGF-A, VEGFR1/2, and ETS-1 were quantified by qRT-PCR; cohort-matched miRNA profiling (microarray with qRT-PCR validation) was integrated. Elevated VEGF-A at tumor depth predicted recurrence (log-rank *P* = 0.0001), whereas surface VEGF-A had no prognostic value (*P* = 0.170). Pairwise testing confirmed higher VEGF-A at depth versus surface (Wilcoxon *P* = 0.026). A subgroup with depth VEGF-A relative quantification (RQ) > 2 and HIF1α RQ < 2 showed a 64% recurrence rate. An independent subgroup defined by high miR-93-5p/miR-144-3p/miR-210-3p expression also had significantly worse outcomes. The two subgroups were non-overlapping in most patients and accounted for 76% of recurrences. The prognostic relevance of VEGF-A in LSCC is region dependent, with clinically meaningful value confined to tumor depth. Together with a high-risk three-miRNA signature, these findings delineate two molecular subgroups capturing most recurrences and may inform sampling strategies and risk stratification.

**Significance:**

We demonstrate that VEGF-A has prognostic value in laryngeal carcinoma only when assessed at tumor depth. Alongside a distinct high-risk miRNA signature, we define two molecular subgroups accounting for most recurrences, highlighting the clinical importance of spatial heterogeneity in biomarker evaluation.

## Introduction

Over the past decade, our research group has been actively engaged in investigating the molecular profile of laryngeal carcinoma, with a primary focus on elucidating the processes of neoangiogenesis and miRNA expression patterns. In the present study, we defined two major objectives: (i) to clarify the ambiguity of vascular endothelial growth factor-A (VEGF-A) as a prognostic marker in laryngeal carcinoma in light of tumor heterogeneity and (ii) to identify distinctive expression phenotypes among major proangiogenic molecules and microRNAs that strongly correlate with recurrence and survival outcomes, thus demarcating independent molecular subgroups of patients.

The combined dataset utilized in this study integrates mRNA and miRNA expression profiles derived from samples collected from our group across various projects dedicated to the exploration of angiogenesis and noncoding RNAs (ncRNA). The prospective design of sample collection enabled us to explore the degree of tumor heterogeneity between the tumor depth and surface, as well as the histologically healthy peritumoral mucosa. Notably, all samples were systematically compared with paired control samples obtained from healthy distant laryngeal mucosa of the same patient. This comprehensive approach not only enhances the statistical robustness of our findings but also ensures the accuracy of fold-change assessments (1).

Tumor angiogenesis is a cornerstone of cancer pathology and is recognized early in cancer pathology as a pivotal process driving tumor progression (2). Its significance extends beyond mere pathogenesis, influencing patient prognosis and shaping the trajectory of cancer treatment strategies. Extensive efforts have been dedicated to unraveling the prognostic implications of key proangiogenic genes, with a particular emphasis on VEGF-A, arguably the most scrutinized among them. Despite the widely acknowledged association between VEGF-A and adverse prognosis, its prognostic reliability remains a subject of contention, marked by inconsistent reproducibility across studies (3). Although higher expression levels of VEGF-A typically correlate with advanced tumor stages, its prognostic relevance becomes ambiguous within more homogeneous stage-defined cohorts. Furthermore, the emergence of ncRNAs, particularly potential biomarkers, has revolutionized the landscape of cancer research. Once considered as transcriptional noise, ncRNAs have garnered significant attention for their regulatory roles in diverse cellular processes, including tumorigenesis. MicroRNAs (miRNA), a class of small ncRNAs, have emerged as key players in posttranscriptional gene regulation and have been implicated in various aspects of cancer biology, including tumor initiation, progression, and metastasis. Their stability in bodily fluids and differential expression patterns in patients with cancer make them promising candidates as noninvasive diagnostic and prognostic markers. Harnessing the potential of ncRNAs as biomarkers holds promise for improving early detection, prognostic stratification, and personalized treatment strategies in patients with cancer.

## Materials and Methods

### Patients and eligibility

A total of 60 patients (mean age at diagnosis, 64.6 ± 8.7 years) with pathologically verified primary laryngeal carcinoma were included in the current prospective prognostic study. Inclusion criteria were patients with T3-/T4-stage laryngeal carcinoma primary tumors. All female patients were at a postreproductive age. Exclusion criteria were any chemo- or radiotherapy prior to surgery. All enrolled participants finished the study, i.e., there was no attrition. Randomization was not applicable and a formal power calculation was not performed. The patients were admitted to the Department of ENT, Head and Neck Surgery, Medical University, Sofia, from 2018 to 2019 as they underwent primary laryngectomy. During surgery, four samples from each patient were obtained: the tumor site surface, tumor site depth, histologically healthy peritumor mucosa within 1 cm from the border of the tumor, and paired normal laryngeal mucosa distant from the tumor (contralateral, at least 3 cm distance). All samples were stored in RNAlater Solutions (Thermo Fisher Scientific), frozen at −20°C for a short period until they were transported to the Molecular Medicine Center, Department of Medical Chemistry and Biochemistry, Medical University, Sofia, and maintained at −80°C until use.

All the patients signed an informed consent form. This study was approved by the Ethics Committee of the Medical University, Sofia. The enrolled cohort was a single-surgeon consecutive series, and the inclusion criteria were described in a previous study (4). All patients underwent postoperative radiotherapy or combined chemoradiotherapy, according to the protocol.

### Anatomic definition of regions

Tumor surface: the most superficial viable layer of the tumor in contact with the lumenal side of the larynx, after removal of gross necrotic debris and mucus.

Tumor depth (core): the deepest viable portion of the tumor, sampled perpendicular to the lumenal surface at the invasive front, avoiding necrotic or cartilaginous areas.

Peritumoral mucosa: histologically normal mucosa within ≤1 cm of the gross tumor edge, oriented away from the resection margin.

Distant normal mucosa (paired control): contralateral laryngeal mucosa ≥3 cm from the tumor.

Sampling design was as follows: From each location (surface, depth, peritumoral mucosa, and distant normal mucosa), a single specimen measuring ≥6 mm in width and 2 to 3 mm in depth was obtained per patient. A minimum interval of ≥5 mm between surface and depth sampling sites was maintained. All specimens were homogenized prior to RNA extraction to ensure regional representativeness.

### RNA extraction

Using the miRNeasy Mini Kit from Qiagen, we isolated total RNA, including miRNAs, from 60 fresh-frozen paired samples consisting of tumor (depth and surface) and adjacent normal tissues. Initial RNA quantification was conducted using a NanoDrop 2000 Spectrophotometer (Thermo Fisher Scientific, RRID: SCR_018042), whereas more accurate measurements were obtained using the Qubit platform via the RNA HS Assay Kit and Qubit 2.0 Fluorometer (Life Technologies, Thermo Fisher Scientific, RRID: SCR_020553). The rigorously determined quantification allowed for the preparation of well-defined RNA concentrations essential for subsequent analyses: 50 ng/μL for miRNA array analysis and 100 ng/μL for qRT-PCR assays. RNA quality was assessed using the Agilent 2100 Bioanalyzer platform (Agilent, RRID: SCR_018043) to determine the RNA integrity number as described previously (4).

### Datasets

The microarray dataset with RT-PCR validation in all 60 patients was previously published (4) and served as the source of normalized miRNA expression data. In the present study, additional RT-PCR analyses of key proangiogenic genes were performed on the same samples. The new gene expression results were integrated with the existing miRNA dataset to enable combined molecular profiling, subgroup analysis, and survival assessment. This approach ensured methodologic consistency and maintained transparent linkage between the datasets (Supplementary Table S1).

### Microarray analysis of miRNAs

MicroRNA expression patterns were analyzed using high-throughput microarray technology. The study employed Agilent’s G3 Human MiRNA Microarray platform (release 21.0, 8 × 60 K) with AMADID no. 070156, following the methodologic procedures detailed in an earlier publication (4).

### qRT-PCR

Gene expression analysis was performed via qRT-PCR using the 7900HT Fast Real-Time PCR System (Applied Biosystems, RRID: SCR_018060). Template cDNA was generated from 400 ng of total RNA using a High-Capacity cDNA RT Kit (Applied Biosystems; Thermo Fisher Scientific) and miScript II RT Kit (Qiagen). The High-Capacity cDNA Kit detects mRNAs, and the miScript II RT Kit was designed for improved miRNA detection. This study targeted key angiogenesis-related transcripts [hypoxia-inducible factor 1α (HIF1α), HIF2α, HIF3α, VEGF-A, VEGFR1, VEGFR2, and ETS-1) and miR-210 using commercially available QuantiTect Primer Assays and miScript Primer Assays (Qiagen). The PCRs were performed in triplicate, with each 10 μL reaction containing SYBR Green PCR Mix (Qiagen) and the respective primer assays. To ensure accurate quantification, the expression levels were normalized to those of endogenous controls: β-actin for mRNA targets and U6 small nuclear RNA for miR-210. The analysis included appropriate negative and no-template controls, and relative quantification (RQ) was determined using the 2^−ΔΔCt^ method, as detailed in a previous study (4).

### IHC

To provide protein-level validation, VEGF-A IHC was performed in a representative cohort of eight patients. In accordance with the sampling protocol of the primary cohort, paired specimens representing the tumor surface and the tumor depth were obtained for each case. Staining was performed using VEGF antibody [VEGF (VG1) antibody, cat. #PDM165 prediluted, Diagnostic Biosystems] and intensity/extent was evaluated by two certified pathologists using the H-score method (0–300; Supplementary Table S2).

### Statistical analysis

The primary endpoint was recurrence-free survival (RFS). Univariable comparisons were performed using Kaplan–Meier survival analysis with log-rank testing, and paired differences between tumor surface and depth were assessed using the Wilcoxon signed-rank test. To account for potential confounding, multivariable Cox proportional hazards models were constructed, adjusting for pT category (T3 vs. T4), nodal status (N0–3), extranodal extension (present/absent), tumor thickness (mm), smoking exposure (pack-years), and human papillomavirus (HPV) status (p16 IHC). The Kolmogorov–Smirnov test for normality and Wilcoxon test were used as appropriate. The Spearman correlation test was used to analyze the relationships between two continuous variables. Statistical significance was defined as a two-tailed *P* value < 0.05. SPSS software (version 23.0; IBM, RRID: SCR_016479) and GraphPad Prism software (RRID: SCR_002798) were used for the data analysis.

## Results

### Clinicopathologic characteristics and survival

The study cohort consisted of 60 cases, with the majority (90.3%) being HPV-negative tumors, as confirmed through p16 IHC. All the patients had a history of long-term smoking. With respect to tumor stage, most cases (87.1%) were classified as pT4a, with one case being pT4b, and the remaining classified as pT3. Nearly half of the patients (48.3%) had a pathologically confirmed metastatic disease. The N status distribution was as follows: N1 (28.6%), N2a/N2b/N2c (57.1%), and N3 (14.3%). A full summary of the clinicopathologic characteristics of the cohort is presented in Table 1.

**Table 1.** Clinicopathologic characteristics of the study cohort.

Characteristic	Number of patients	Percentage
Age	64 (mean), 46–83	N/A
Gender	58 males and 2 females	96.6% vs. 3.3%
T-stage	​	​
pT3	7	11.6%
pT4a and pT4b	53	88.3%
*N*-stage	​	​
pN0	32	53.3%
pN1	7	11.6%
pN2a	2	3.3%
pN2b	8	13.3%
pN2c	7	11.6%
pN3	4	6.6%
Metastasis (pN0 vs. pN1–3)	​	​
pN0	32	53.3%
pN1–3	28	46.7%
Grade	​	​
G1	15	25%
G2	42	70%
G3	3	5%

### VEGF-A expression in tumor depth strongly predicted recurrence, whereas surface expression and other proangiogenic markers showed no prognostic value

**Figure 1.**
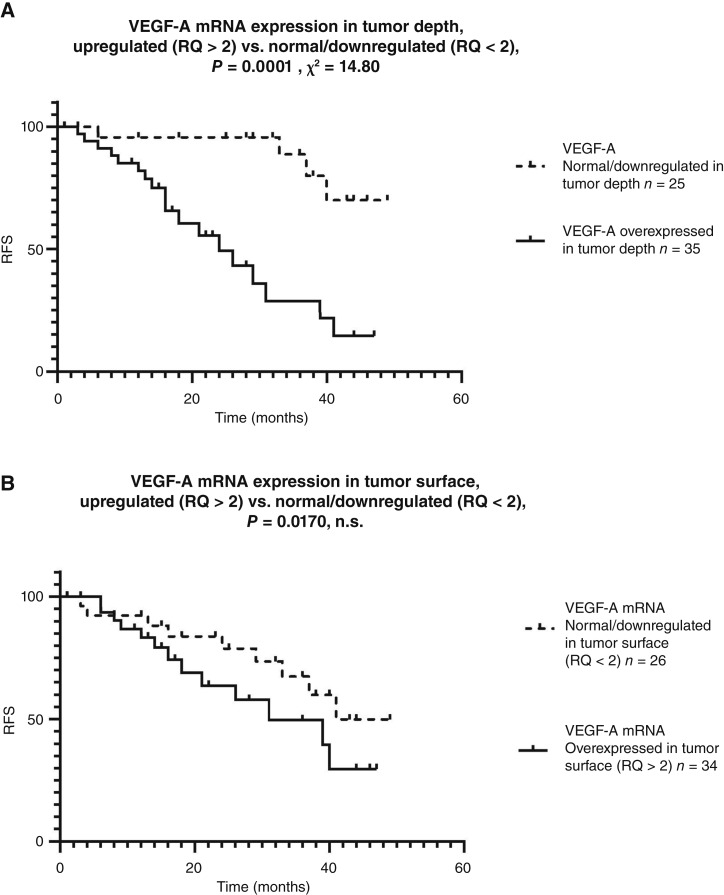
Kaplan–Meier RFS (median follow-up of 24 months). **A,** Patients with elevated VEGF-A at tumor depth (RQ > 2; *n* = 35; mean = 7.06 ± 5.38) had significantly worse outcomes than those with lower expression (RQ ≤ 2; *n* = 25; mean = 1.17 ± 0.48; log-rank *P* = 0.0001 and *χ*^2^ = 14.8). **B,** VEGF-A expression at the tumor surface was not prognostic (log-rank *P* = 0.170).

We analyzed the mRNA expression levels of major proangiogenic molecules on the tumor surface and tumor depth, namely HIF1α, HIF2α, HIF3α, VEGF-A, VEGFR1, VEGFR2, and ETS-1. We did not find any statistically significant associations between any of these molecules and the recurrence rate, with one exception: the mRNA expression levels of VEGF-A were measured at greater depths in the tumor. Patients with upregulated levels of VEGF-A (RQ > 2; *n* = 35; mean = 7.06 ±5.38) in terms of tumor depth had a worse prognosis than those with normal or downregulated (RQ < 2; *n* = 25; mean = 1.17 ±0.48) expression levels (log-rank *P* = 0.0001, *χ*^2^ = 14.8; Fig. 1A). In contrast, VEGF-A expression levels measured in tumor surface among the same group of patients were not associated with survival (log-rank *P* = 0.170; Fig. 1B), regardless of almost identical subgroup characteristics: the VEGF-A tumor depth subgroup with RQ > 2: *n* = 35 and mean = 7.05 ±5.38 versus the VEGF-A tumor surface subgroup with RQ > 2: *n* = 34 and mean = 7.17 ± 10.8. To evaluate the expression patterns of VEGF-A between the two tumor subsites more thoroughly, we conducted additional parallel analyses.

In multivariable Cox regression, including depth VEGF-A expression and primary tumor size measured on CT, both variables were associated with RFS. Depth VEGF-A expression (RQ > 2) conferred a 4.7-fold increased hazard of recurrence [HR = 4.68; 95% confidence interval (CI), 1.01–21.64; *P* = 0.048], independent of tumor size. Each 1-cm increase in CT-measured tumor dimension was associated with a 1.5-fold increase in recurrence hazard (HR = 1.50; 95% CI, 1.00–2.24; *P* = 0.050).

### VEGF-A mRNA expression was higher at tumor depth than surface only in paired analyses, indicating relative rather than absolute heterogeneity across patients

**Figure 2.**
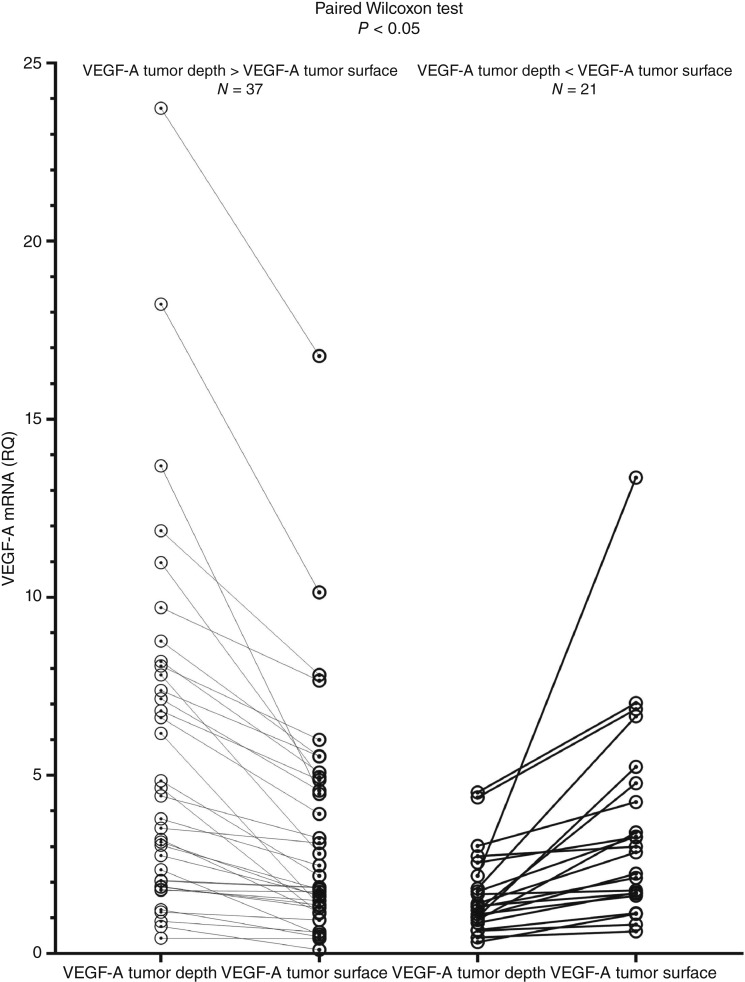
Paired analysis demonstrated significantly higher VEGF-A mRNA expression at tumor depth compared with surface (Wilcoxon *z* = −2.219; *P* = 0.026).

Analysis of the mRNA expression profile of VEGF-A revealed similar characteristics in terms of tumor depth and surface area: the mean RQ at the tumor depth for the whole group was 4.48 ± 4.99 versus 3.56 ± 3.20 at the tumor surface; the median RQ values were 2.54 and 2.47, respectively. When the analysis was run with an unpaired nonparametric test (Mann–Whitney U test), no statistically significant difference was found between the two expression profiles. However, when both sample groups were compared in a paired fashion using the nonparametric Wilcoxon paired test, a statistically significant difference was observed. The difference scores were approximately symmetrically distributed as assessed using a histogram with a superimposed normal curve. VEGF-A mRNA expression levels were significantly higher at greater depths than at the tumor surface (*z* = −2.219; *P* = 0.026; Fig. 2). These findings provide evidence for the existence of statistically significant VEGF-A heterogeneity in advanced laryngeal carcinoma, which is not absolute in terms of RQ values but is relative for each individual patient. Tumor depth is associated with higher levels of VEGF-A mRNA expression than surface depth.

### IHC validation

**Figure 3.**
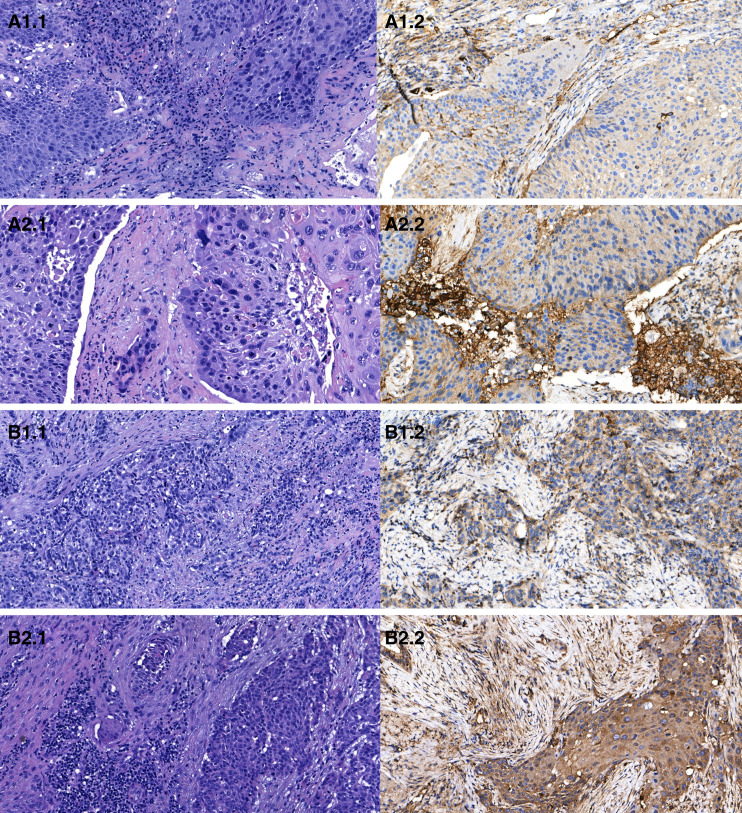
Representative hematoxylin and eosin and IHC staining from two cases. Case 3: (**A1.1**) hematoxylin and eosin of tumor surface, (**A1.2**) VEGF-A IHC at tumor surface, (**A2.1**) hematoxylin and eosin of tumor depth, and (**A2.2**) VEGF-A IHC at tumor depth, showing no qualitative differences between regions. Case 5: (**B1.1**) hematoxylin and eosin of tumor surface, (**B1.2**) VEGF-A IHC at tumor surface, (**B2.1**) hematoxylin and eosin of tumor depth, and (**B2.2**) VEGF-A IHC at tumor depth, with a slight tendency toward stronger expression in the depth.

VEGF-A IHC was assessed in eight representative cases sampled analogously to the primary cohort. No consistent qualitative differences between surface and depth were observed, and semiquantitative H-scores did not differ significantly (paired-samples *T* test, mean ± SD: 177.9 ± 28.4 vs. 171.6 ± 36.2; *P* = 0.702; Fig. 3; Supplementary Table S2). Isolated cases showed a possible trend toward stronger staining at depth, but this did not reach significance. Within the limits of this small set and the semiquantitative nature of IHC, depth-restricted heterogeneity evident at the mRNA level was not demonstrable at the protein level.

### A subgroup with depth VEGF-A upregulation (RQ > 2) and HIF1α downregulation (RQ < 2) showed markedly higher recurrence rates

**Figure 4.**
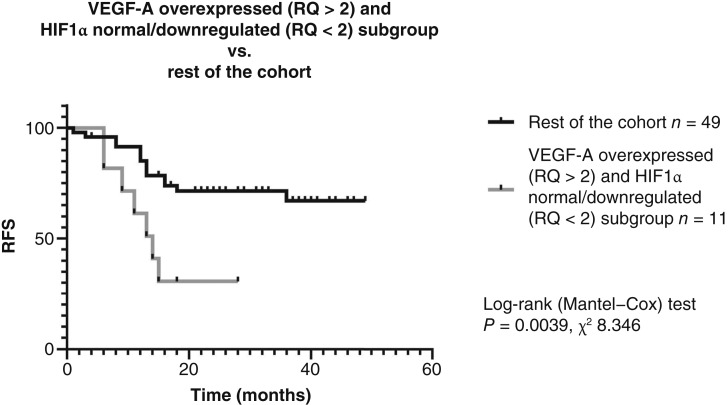
Subgroup of patients with VEGF-A upregulation at depth (RQ > 2) and HIF1α downregulation (RQ ≤ 2) showed markedly higher recurrence rates (log-rank *P* = 0.0039; *χ*^2^ = 8.346).

After a deeper analysis of the proangiogenic expression profile in laryngeal carcinoma, we identified a specific subgroup of patients with overexpression of VEGF-A (RQ > 2) and normal/downregulated expression of HIF1α (RQ < 2). This subgroup consisted of 11 patients (18.3% of the whole cohort), and among these patients, seven experienced recurrence during the follow-up period, which represents a 64% recurrence rate, whereas 28.5% of the remaining patients experienced recurrence (Pearson *χ*^2^ test, *P* = 0.028). The overall recurrence rate of the entire cohort was 35%. Additionally, nine of 11 patients had metastasis—82% versus 40.8% for the whole cohort (Pearson *χ*^2^*P* = 0.014). Survival analysis with Kaplan–Meier curves also confirmed a significant difference for this subgroup in terms of the recurrence rate, with log-rank *P* = 0.0039 and *χ*^2^(2) = 8.346 (Fig. 4).

### A second subgroup with high co-expression of miR-93-5p, miR-144-3p, and miR-210-3p had significantly worse prognosis

**Figure 5.**
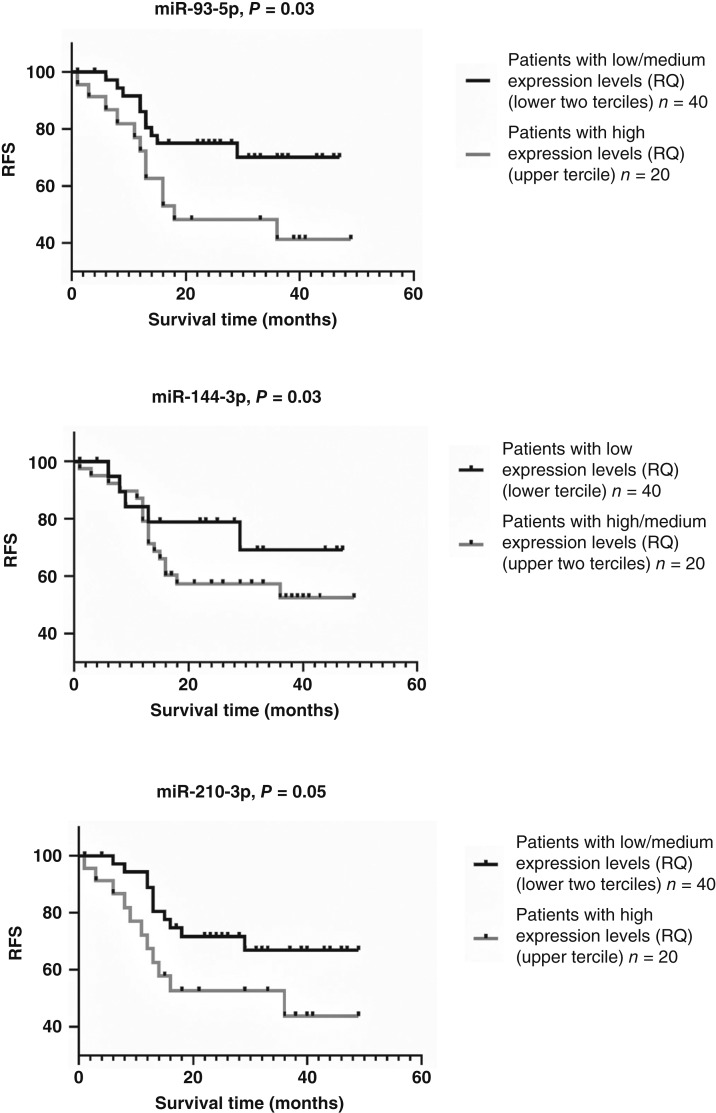
High expression of miR-93-5p, miR-210-3p, and miR-144-3p was associated with significantly reduced RFS [*χ*^2^(2) = 4.68, log-rank *P* = 0.03; *χ*^2^(2) = 4.53, log-rank *P* = 0.03; and *χ*^2^(2) = 4.53, log-rank *P* = 0.03, respectively]. Previously published in ref. 4.

In 2022, our group published a study reporting a global miRNA microarray expression profile in advanced laryngeal carcinoma (4). After RT-PCR validation, three miRNAs, namely, miR-93-5p, miR-144-3p, and miR-210-3p, tended to be strong predictors of the recurrence of advanced laryngeal carcinoma (Fig. 5, published previously). Patients were divided into tertiles based on RQ expression: low, medium, and high, following methodologies from similar studies. Kaplan–Meier analysis indicated that patients with high expression levels of the oncogenes miR-93-5p, miR-210-3p, and miR-144-3p had significantly worse survival rates than those with low or medium expression levels [*χ*^2^(2) = 4.68, log-rank *P* = 0.03; *χ*^2^(2) = 4.53, log-rank *P* = 0.03; and *χ*^2^(2) = 4.53, log-rank *P* = 0.03, respectively]. Spearman rank-order correlation was used to assess the relationship between each of the three miRNAs; all three molecules exhibited strong correlations with each other, which revealed co-expression associations between the three molecules.

### The VEGF-A–/HIF1α- and miRNA-defined subgroups were independent of one another and collectively accounted for 76% of recurrences in the cohort

We performed an integrative analysis combining gene and microRNA expression data obtained from the same cohort of 60 patients. The microarray dataset, with RT-PCR validation in all patients, was previously published as stated before (4) and served as the source of normalized miRNA expression data. In the present study, additional RT-PCR analyses of key proangiogenic genes were performed on the same samples. The new gene expression results were then integrated with the existing miRNA dataset to enable combined molecular profiling, subgroup analysis, and survival assessment, ensuring methodologic consistency and transparent linkage between the datasets (Supplementary Table S1).

**Figure 6.**
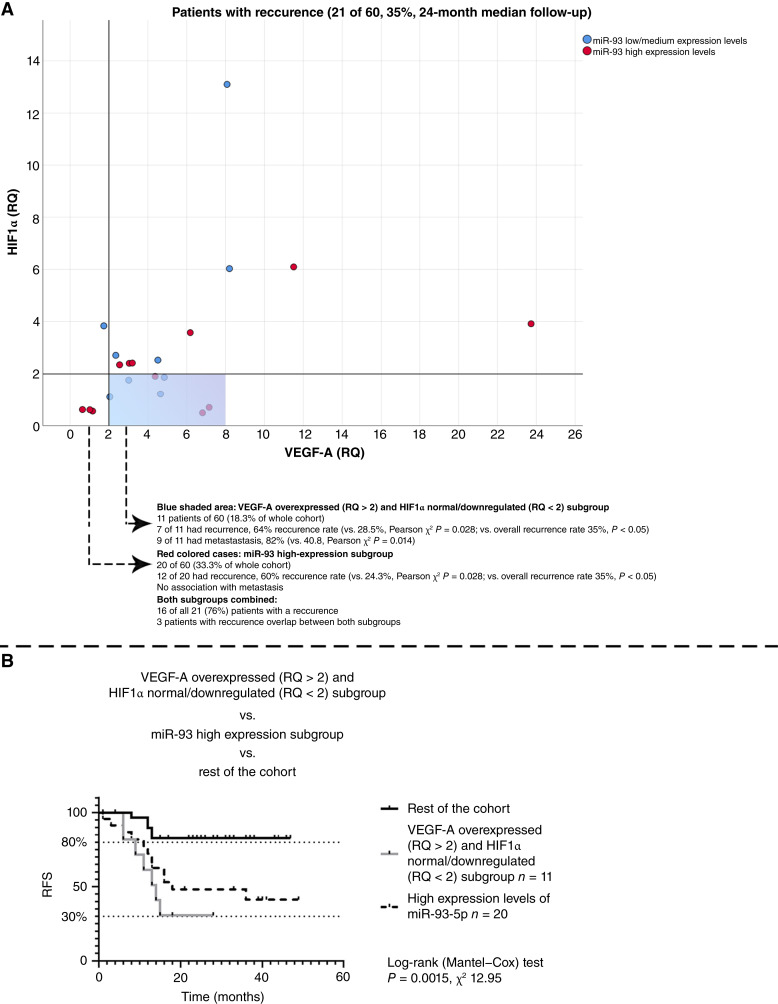
**A,** Distribution of recurrent cases by molecular subgroup. The VEGF-A/HIF1α subgroup (blue) and the miRNA co-expression subgroup (red) were independent and together accounted for 76% of recurrences. **B,** Kaplan–Meier analysis confirmed significantly poorer outcomes for both subgroups (log-rank *P* = 0.0015; *χ*^2^ = 12.95).

No significant correlation or other form of association was observed between VEGF-A and the three co-expressed miRNAs, confirming the independence of their expression patterns. Analysis was then directed toward the 21 patients (35% of the total cohort) who developed tumor recurrence. Among these, seven were part of the VEGF-A (RQ > 2)/HIF1α (RQ < 2) subgroup (seven of 11; 64%; Pearson *χ*^2^*P* = 0.028) and 12 belonged to the miR-93-5p/miR-144-3p/miR-210-3p subgroup (12 of 20; 60%; Pearson *χ*^2^*P* = 0.028). Three patients overlapped and were affiliated with both subgroups (Fig. 6A). Overall, 16 of 21 patients with recurrence (76.2%) belonged to one of the two independent subgroups, whereas five patients (23.8%) were not associated with either distinctive expression pattern. Kaplan–Meier analysis demonstrated a significant difference in survival among these subgroups [log-rank test, *P* = 0.0015; *χ*^2^(2) = 12.95; Fig. 6B].

## Discussion

In this study, we integrated mRNA and miRNA expression data with RFS in a single prospective cohort of advanced laryngeal carcinoma. Four principal findings emerged. First, VEGF-A expression was prognostic only when assessed at tumor depth, whereas surface expression lacked predictive value. Second, VEGF-A heterogeneity between surface and depth was relative rather than absolute, varying within individual patients. Third, a distinct subgroup characterized by downregulated HIF1α (RQ < 2) and concomitant upregulation of VEGF-A at depth (RQ > 2) exhibited a markedly elevated recurrence rate (64%). Fourth, an independent subgroup defined by high co-expression of miR-93-5p, miR-144-3p, and miR-210-3p had a recurrence rate of 60%. Collectively, these two molecularly defined subgroups accounted for approximately 76% of recurrences in the cohort.

In contrast to the mRNA analyses, VEGF-A IHC in a small validation cohort did not demonstrate significant qualitative or semiquantitative differences between tumor surface and depth. This discrepancy may reflect the inherent limitations of IHC, which provides only semiquantitative information and lacks the sensitivity of qRT-PCR for detecting subtle fold changes in gene expression. Additionally, a divergence between VEGF-A mRNA expression and corresponding protein staining may reflect posttranscriptional regulatory mechanisms that influence protein synthesis, stability, or secretion independently of transcript abundance. Such regulation can lead to differences between RNA-based and protein-based measurements, even when both are biologically valid. Thus, although the IHC results did not replicate the mRNA findings, future validation using more advanced spatial proteomic methods would be necessary. In addition, multisite sampling or heterogeneity assessment strategies may represent a practical means to improve biomarker reliability as they could capture depth-restricted VEGF-A expression that single superficial samples might miss.

VEGF-A has been extensively studied across different tumor types, including laryngeal carcinoma, yet its role as a predictor of recurrence remains controversial. Although numerous reports have described an association between elevated VEGF-A expression and poorer survival outcomes, several well-conducted studies have questioned the reproducibility of such findings (5). One important limitation of the published literature is that most cohorts are stage heterogeneous, typically combining cases from T1 to T4, for which prognosis is largely driven by tumor stage itself rather than molecular markers. This stage dependence complicates interpretation of VEGF-A’s prognostic role. By contrast, the present study was restricted to advanced, stage-homogeneous tumors, with more uniform tumor size and extent, thereby minimizing stage as a confounding factor and allowing a clearer assessment of molecular predictors.

To the best of our knowledge, our study is the first to explore heterogeneity in laryngeal carcinoma. Our findings revealed that absolute expression levels are not fundamentally valuable, but a more important characteristic is the expression pattern of VEGF-A in certain locations of the tumor. One possible explanation for the observed heterogeneity in VEGF-A expression is its relationship to the spatial distribution of cancer stem cell populations within the tumor as described by Fotinós and colleagues (6). In addition to its canonical angiogenic role, VEGF-A has been implicated in stem cell–related processes such as epithelial–mesenchymal transition (7). Recent insights also highlight VEGF signaling’s broader impact on tumor biology, including immune suppression and metabolic cross-talk within the microenvironment, which may influence therapeutic resistance (8). Zhang and colleagues (9) further suggested that VEGF-A may mediate cancer cell stemness via the VEGF/NRP2–Hippo pathway axis. Under hypoxic conditions, HIF1α typically drives VEGF-A transcription via hypoxia-responsive elements in its promoter (10), making VEGF-A a key effector of the canonical HIF pathway. The subgroup in our cohort with elevated VEGF-A despite normal or downregulated HIF1α suggests a possible activation via nonhypoxic mechanisms. If so, the prognostic impact of VEGF-A in this subgroup may reflect a stemness-associated role rather than its traditional proangiogenic function, a hypothesis that warrants further functional validation. Recognizing this distinction is important as it may have potential clinical implications, particularly in the context of VEGF pathway inhibition strategies currently under investigation in head and neck cancer.

A limitation of the present work is that VEGF-A IHC in a representative validation cohort did not reproduce the depth–surface differences observed at the mRNA level. Given the semiquantitative nature and lower sensitivity of IHC compared with qRT-PCR, subtle fold changes are unlikely to be reliably detected by this method. This finding underscores the need for validation using larger cohorts and more sensitive spatially resolved proteomic approaches.

### Conclusion

The prognostic value of VEGF-A in laryngeal squamous cell carcinoma depends on spatial context rather than absolute levels, with depth-specific upregulation consistently linked to recurrence. These findings support spatially informed sampling and incorporation of intratumoral heterogeneity into biomarker evaluation.

## Supplementary Material

Supplementary Table 1 Supplementary Table S1. Distribution of samples across analytical techniques

Supplementary Table 2 Supplementary Table 2. H-scores for VEGF Expression in Individual Tumor Samples.

## Data Availability

Raw data were generated at the Medical University, Sofia. The derived data supporting the findings of this study are available online at https://doi.org/10.5281/zenodo.17842777 or upon request from the corresponding author.
