# Supplementary material for: Two Molecular Subgroups Predict Most Recurrences in Advanced Laryngeal Squamous Cell Carcinoma
Source: Cancer Res Commun. 2026 Jan 8;6(1):60–9. doi: 10.1158/2767-9764.CRC-25-0249 (PMC12780839; doi:10.1158/2767-9764.CRC-25-0249)
Supplement: Supplementary Table 1 — Supplementary Table S1. Distribution of samples across analytical techniques [file crc-25-0249_supplementary_table_1_supps1.docx]

**Supplementary Table 1. Distribution of samples across analytical techniques.**

| Analysis type | Number of patients (n = 60) | Description / Notes |
| --- | --- | --- |
| miRNA microarray + RT-PCR validation | 60 (100 %) | Previously published dataset (*Popov et al.*, *Scientific Reports*, 2022 [4]); provided normalized miRNA expression values validated by RT-PCR. |
| RT-PCR for pro-angiogenic genes | 60 (100 %) | Newly performed in the present study on the same RNA samples to quantify VEGF-A, HIF-1α, and other key pro-angiogenic genes. |
| Integrative molecular analysis | 60 (100 %) | Combined dataset including miRNA and gene expression results used for subgroup and survival analyses. |
