# Supplementary material for: Two Molecular Subgroups Predict Most Recurrences in Advanced Laryngeal Squamous Cell Carcinoma
Source: Cancer Res Commun. 2026 Jan 8;6(1):60–9. doi: 10.1158/2767-9764.CRC-25-0249 (PMC12780839; doi:10.1158/2767-9764.CRC-25-0249)
Supplement: Supplementary Table 2 — H-scores for VEGF Expression in Individual Tumor Samples. [file crc-25-0249_supplementary_table_2_supps2.docx]

**Supplementary Table 2. H-scores for VEGF Expression in Individual Tumor Samples.**

|  | 0 | 1+ | 2+ | 3+ | H | 0 | 1+ | 2+ | 3+ | H |
| --- | --- | --- | --- | --- | --- | --- | --- | --- | --- | --- |
|  | 5 | 35 | 50 | 10 | 165 | 40 | 40 | 15 | 5 | 85 |
|  | 10 | 60 | 15 | 15 | 135 | 10 | 40 | 40 | 10 | 150 |
| I | 5 | 20 | 65 | 10 | 180 | 50 | 30 | 18 | 2 | 72 |
|  | 10 | 10 | 60 | 20 | 190 | 0 | 15 | 25 | 60 | 245 |
|  | 20 | 45 | 30 | 5 | 120 | 1 | 19 | 40 | 40 | 219 |
|  |  |  |  |  | 158 |  |  |  |  | 154,2 |
|  | 20 | 20 | 50 | 10 | 150 | 10 | 45 | 35 | 10 | 145 |
|  | 10 | 30 | 40 | 20 | 170 | 0 | 40 | 40 | 20 | 180 |
| II | 10 | 40 | 40 | 10 | 150 | 15 | 35 | 40 | 20 | 175 |
|  | 0 | 35 | 45 | 20 | 185 | 10 | 20 | 50 | 10 | 150 |
|  | 5 | 15 | 40 | 40 | 215 | 15 | 40 | 20 | 25 | 155 |
|  |  |  |  |  | 174 |  |  |  |  | 161 |
|  | 10 | 0 | 20 | 70 | 250 | 5 | 10 | 5 | 80 | 260 |
|  | 10 | 5 | 15 | 70 | 245 | 10 | 50 | 20 | 10 | 120 |
| III | 10 | 10 | 20 | 60 | 230 | 0 | 65 | 15 | 30 | 185 |
|  | 0 | 10 | 20 | 70 | 260 | 5 | 40 | 10 | 40 | 180 |
|  | 10 | 20 | 30 | 40 | 200 | 10 | 40 | 10 | 40 | 180 |
|  |  |  |  |  | 237 |  |  |  |  | 185 |
|  | 5 | 30 | 5 | 60 | 220 | 5 | 64 | 30 | 1 | 127 |
|  | 5 | 65 | 10 | 20 | 145 | 10 | 50 | 35 | 5 | 135 |
| IV | 35 | 40 | 5 | 20 | 110 | 10 | 30 | 40 | 20 | 170 |
|  | 0 | 0 | 20 | 80 | 280 | 0 | 60 | 20 | 20 | 160 |
|  | 0 | 10 | 20 | 70 | 260 | 5 | 25 | 30 | 50 | 235 |
|  |  |  |  |  | 203 |  |  |  |  | 165,4 |
|  | 0 | 39 | 60 | 1 | 162 | 35 | 60 | 2 | 3 | 73 |
|  | 0 | 40 | 60 | 0 | 160 | 40 | 50 | 10 | 0 | 70 |
| V | 0 | 37 | 60 | 3 | 166 | 5 | 60 | 20 | 15 | 145 |
|  | 0 | 30 | 70 | 0 | 170 | 20 | 70 | 5 | 5 | 95 |
|  | 0 | 30 | 60 | 10 | 180 | 10 | 70 | 20 | 0 | 110 |
|  |  |  |  |  | 167,6 |  |  |  |  | 98,6 |
|  | 0 | 30 | 60 | 20 | 210 | 0 | 40 | 30 | 15 | 145 |
|  | 10 | 40 | 40 | 30 | 210 | 5 | 50 | 45 | 45 | 275 |
| VI | 25 | 35 | 15 | 20 | 125 | 10 | 40 | 30 | 25 | 175 |
|  | 20 | 40 | 15 | 0 | 70 | 20 | 35 | 25 | 15 | 130 |
|  | 10 | 35 | 40 | 20 | 175 | 10 | 15 | 25 | 55 | 230 |
|  |  |  |  |  | 158 |  |  |  |  | 191 |
|  | 10 | 55 | 40 | 20 | 195 | 20 | 35 | 70 | 25 | 250 |
|  | 0 | 40 | 15 | 30 | 160 | 20 | 20 | 30 | 40 | 200 |
| VII | 15 | 35 | 15 | 45 | 200 | 5 | 45 | 40 | 55 | 290 |
|  | 15 | 15 | 0 | 40 | 135 | 15 | 25 | 40 | 15 | 150 |
|  | 25 | 15 | 40 | 25 | 170 | 20 | 25 | 50 | 15 | 170 |
|  |  |  |  |  | 172 |  |  |  |  | 212 |
|  | 10 | 20 | 60 | 10 | 170 | 0 | 10 | 30 | 60 | 250 |
|  | 10 | 30 | 40 | 20 | 170 | 0 | 20 | 40 | 40 | 220 |
| VIII | 30 | 10 | 40 | 20 | 150 | 10 | 40 | 20 | 30 | 170 |
|  | 20 | 40 | 30 | 10 | 130 | 5 | 20 | 70 | 5 | 175 |
|  | 10 | 30 | 60 | 0 | 150 | 5 | 15 | 40 | 40 | 215 |
|  |  |  |  |  | 154 |  |  |  |  | 206 |
|  |  |  |  |  |  |  |  |  |  |  |
